# Cardiovascular Death Risk in Primary Central Nervous System Lymphoma Patients Treated With Chemotherapy: A Registry-Based Cohort Study

**DOI:** 10.3389/fonc.2021.641955

**Published:** 2021-05-11

**Authors:** Tianwang Guan, Zicong Qiu, Miao Su, Jinming Yang, Yongshi Tang, Yanting Jiang, Dunchen Yao, Yanxian Lai, Yanfang Li, Cheng Liu

**Affiliations:** ^1^ Department of Cardiology, Guangzhou First People’s Hospital, Guangzhou Medical University, Guangzhou, China; ^2^ Department of Cardiology, Guangzhou First People’s Hospital, South China University of Technology, Guangzhou, China; ^3^ Department of Cardiology, Laboratory of Heart Center, Zhujiang Hospital, Southern Medical University, Guangzhou, China; ^4^ Department of Clinical Medicine, Clinical Medical School, Guangzhou Medical University, Guangzhou, China; ^5^ Department of Oncology, Guiqian International General Hospital, Guiyang, China

**Keywords:** chemotherapy, cardiovascular death, primary central nervous system lymphoma, non-central nervous system lymphoma, SEER, cardio-oncology

## Abstract

**Purpose:**

To study the cardiovascular death (CVD) risk in primary central nervous system lymphoma (PCNSL) patients with chemotherapy.

**Methods:**

We obtained 2,020 PCNSL participants and 88,613 non-central nervous system lymphoma (NCNSL) participants with chemotherapy from Surveillance, Epidemiology, and End Results (SEER) database from 2004 to 2015. A 1:3 propensity score matching (PSM) was used to reduce the imbalance between PCNSL participants with and without chemotherapy, as well as the imbalance between PCNSL and NCNSL participants with chemotherapy. Competing risks regressions were conducted to evaluate the independent influence of chemotherapy on CVD.

**Results:**

After 1:3 PSM, the CVD risk in PCNSL patients with chemotherapy was lower than those without chemotherapy [decreased 53%, adjusted HR, 0.469 (95% CI, 0.255–0.862; *P* = 0.015)] as well as NCNSL patients with chemotherapy [decreased 36%, adjusted HR in model 1, 0.636 (95% CI, 0.439–0.923; *P* = 0.017)]. The CVD risk of chemotherapy decreased in PCNSL patients with age at diagnosis >60 years old [adjusted HR, 0.390 (95% CI, 0.200–0.760; *P* = 0.006)], and those patients diagnosed at 2010 to 2015 [adjusted HR, 0.339 (95% CI, 0.118–0.970; *P* = 0.044)].

**Conclusion:**

PCNSL patients with chemotherapy are associated with lower CVD risk. Our findings may provide new foundations for that chemotherapy is the first-line treatment for PCNSL patients, according to a cardiovascular risk perspective.

## Introduction

Primary central nervous system lymphoma (PCNSL) is a rare but highly aggressive extranodal non-Hodgkin lymphoma (NHL) confined to the central nervous system (1), and the 5- and 10-year survival rates of PCNSL are only 29.9% and 22.2%, respectively (2). The patients with NHL are part of whom are most likely to die of non-cancer deaths, among which cardiovascular death (CVD) is the leading cause of non-cancer deaths (3). As for PCNSL patients, reducing the CVD risks may help improve their prognosis. Therefore, the CVD risks in patients with PCNSL should not be neglected.

Currently, chemotherapy is recommended as the first-line treatment for PCNSL patients, which has advanced in the last decades and involves multiple agents, such as methotrexate (MTX), cytarabine, rituximab carmustine, and so on (4, 5). Generally, chemotherapy is considered to induce cardiotoxicity and increase CVD risk (6). Since high dose of chemotherapy drug is needed for PCNSL chemotherapy to penetrate the blood-brain barrier, the cardiotoxicity may be worse. However, the influence of chemotherapy on CVD in PCNSL patients is still controversial. On the one hand, cytarabine and carmustine can induce cardiotoxicity. Several case reports showed that cytarabine could induce pericarditis (7, 8). Kang et al. found that carmustine impaired systolic function by inhibiting mitochondrial glutathione reductase and inducing mitochondrial dysfunction (9). On the other hand, MTX is considered to improve cardiovascular disease. A systematic review and meta-analysis reported that MTX was related to 21% lower risk for total cardiovascular events and 18% lower risk of myocardial infarction (10). Whether chemotherapy increases the CVD risk or not in PCNSL patients is still ambiguous, which needs to be investigated further.

Hence, a registry-based cohort study was conducted to study the CVD risk in PCNSL patients with chemotherapy, which may contribute to improving prognosis of PCNSL patients and assist clinical decision-making.

## Methods

### Data Source

The data for this study were obtained from 18 cancer registries of Surveillance, Epidemiology, and End Results (SEER) database from 2004 to 2015. The SEER database is an authoritative data system covering about 34.6% of American population. Ethical approval was not required for publicly available information.

### Study Population

PCNSLs was defined as patients with a first malignant PCNSL. The selection criteria were as follow: (1) case selection (Site and Morphology. Histology recode-Brain grouping) = “Lymphoma”; (2) Histology diagnosed as PCNSL. The exclusion criteria were as follows: (1) participants who received radiotherapy; (2) participants with multiple primary tumors; (3) either autopsy only or death certificate only; (4) unknown race; (5) unknown marital status. Finally, a total of 2,020 PCNSL participants were extracted according to the selection and exclusion criteria.

In addition, non-central nervous system lymphoma (NCNSL) patients were defined as patients with a first primary malignant NHL outside central nerve system, which were set as another control group. A total of 88,613 NCNSL patients with chemotherapy were also extracted according to the selection and exclusion criteria (**Supplementary Methods**).

### Participant Variables and Outcomes

PCNSL participants were classified into two groups: chemotherapy and no evidence, based on the chemotherapy selection. Participants’ variables included age at diagnosis (≤60 years, >60 years), sex (male, female), race (white, black, others), marital status (married, unmarried), year of diagnosis (2004–2009, 2010–2015), tumor location (brain, spine, not specific primary site of central nerve system), histological type (mature B-cell NHL, others), and surgery (yes, no evidence). In this study, CVD was the primary endpoint which was defined as the time from the diagnosis of PCNSL to the death from cardiovascular disease. According to the International Classification of Diseases-10 (ICD-10) codes, CVD includes disease of heart, hypertension without heart disease, cerebrovascular disease, atherosclerosis, aortic aneurysm and dissection, and other diseases of arteries, arterioles, and capillaries (11, 12). Censored observations were defined as participants living at the time of last follow-up or dying from non-CVD.

### Statistical Analysis

Categorical variables in baseline characteristics were compared using Chi-square test. A 1:3 propensity score matching (PSM) which was calculated with logistic regression was used for the imbalance between chemotherapy and no evidence groups. PSM should adjust for the potential confounding variables instead of all baseline variables (13). The potential confounding variables were enrolled into the propensity score calculation: age at diagnosis, sex, year of diagnosis, tumor location, histological type, and surgery. The match was conducted using nearest-neighbor algorithm with caliper width of 0.02. A *P* value of larger than 0.05 for the above covariates was regarded as acceptable balance (14). Although there are no sufficient cases for pairing, the 1:3 PSM is still acceptable for making good use of the data (15, 16). The univariate and multivariate Fine and Gray’s competing risks regressions were employed to evaluate the independent effect of chemotherapy on CVD in PCNSL (17).

In addition, the 1:3 propensity score matching (PSM) also was applied to reduce the imbalance between PCNSL patients with chemotherapy and NCNSL patients with chemotherapy (**Supplementary Methods**). The univariate and multivariate Fine and Gray’s competing risks regressions were also utilized to evaluate the effect of chemotherapy on CVD risk between PCNSL and NCNSL. It is a common and widely accepted method that other-site tumor is used as a comparison group (18, 19).

SPSS version 25.0 (SPSS, Chicago, IL) was used to analyze test. R software version 3.6.1 (https://www.r-project.org) was used to conduct PSM. Stata version 15 (StataCorp, College Station, TX, USA) was utilized to perform Fine and Gray’s competing risks regression. Hazard ratios (HRs) were reported within ninety-five percent confidence intervals (95% CIs). Statistical significance was defined by a two-tailed *P* value less than 0.05.

## Results

### Baseline Characteristics

Among 2,020 PCNSL patients, 1,493 (73.9%) received chemotherapy and 527 (26.1%) did not (**Table 1**). Before PSM, chemotherapy group was prone to be younger and had more Mature B-cell NHL patients. After the 1:3 PSM, 1,831 patients were allocated to the matched cohort, and the confounding covariates (age at diagnosis, sex, year of diagnosis, tumor location, histological type, and surgery) were well balanced between the chemotherapy and no chemotherapy groups (**Table 1**). Other covariates (marital status and race) were unbalanced between two groups (**Table 1**), but had no confounding effect on chemotherapy and CVD (**Tables 2** and **S4**). The average follow-up time was 26.6 months in PCNSL patients.

**Table 1:** Baseline characteristics before and after propensity score matching in PCNSLs.

Variable	Before PSM (N/%)			After PSM (N/%)		
	No evidence	CT	*P* value	No evidence	CT	*P* value
**N**	527 (26.1)	1493 (73.9)		527 (28.8)	1304 (71.2)	
**Age at diagnosis**			**0.010**			0.209
≤60 years	192 (36.4)	642 (43.0)		192 (36.4)	518 (39.7)	
>60 years	335 (63.6)	851 (57.0)		335 (63.6)	786 (60.3)	
**Sex**			0.693			1.000
Male	282 (53.5)	784 (52.5)		282 (53.5)	698 (53.5)	
Female	245 (46.5)	709 (47.5)		245 (46.5)	606 (46.5)	
**Race**			**<0.001**			**<0.001**
White	396 (75.1)	1207 (80.8)		396 (75.1)	1057 (81.1)	
Black	66 (12.5)	90 (6.0)		66 (12.5)	78 (6.0)	
Others^#^	65 (12.3)	196 (13.2)		65 (12.3)	169 (13.0)	
**Marital status**			**<0.001**			**<0.001**
Married	266 (50.5)	931(62.4)		266 (50.5)	800 (61.3)	
Unmarried	261 (49.5)	562 (37.6)		261 (49.5)	504 (38.7)	
**Year of diagnosis**			0.268			0.507
2004–2009	253 (48.0)	675 (45.2)		253 (48.0)	602 (46.2)	
2010–2015	274 (52.0)	818 (54.8)		274 (52.0)	702 (53.8)	
**Tumor location**			0.085			0.913
Brain	475 (90.1)	1290 (86.4)		475 (90.1)	1181 (90.6)	
Spine	14 (2.7)	57 (3.8)		14 (2.7)	36 (2.8)	
NOS*	38 (7.2)	146 (9.8)		38 (7.2)	87 (6.7)	
**Histological Type**			**0.036**			0.155
Mature B-cell NHL	453 (86.0)	1334 (89.4)		453 (86.0)	1154 (88.5)	
Others^$^	74 (14.0)	159 (10.6)		74 (14.0)	150 (11.5)	
**Surgery**			0.431			0.816
Yes	212 (40.2)	863 (57.8)		212 (40.2)	534 (41.0)	
No evidence	315 (59.8)	630 (42.2)		315 (59.8)	770 (59.0)	

^#^Others include American Indian/Alaska Native and Asian/Pacific Islander.

*Not specific primary site of central nerve system.

^$^Others include peripheral T-cell lymphoma, anaplastic large cell lymphoma, extranodal NK-/T-cell lymphoma, blastic plasmacytoid dendritic cell neoplasm, precursor B-lymphoblastic lymphoma, precursor T-cell lymphoblastic lymphoma and not specific non-Hodgkin’s lymphoma. The bold values mean P value < 0.05.

CT, Chemotherapy; NOS, not otherwise specific; PSM, propensity score matching.

**Table 2:** Univariate competing-risks regression analysis of cardiovascular death in PCNSLs.

Variable	Before PSM		After PSM	
	HR (95% CI)	*P* Value	HR (95% CI)	*P* Value
**Chemotherapy**		**0.014**		**0.010**
Yes	0.476 (0.262–0.862)	**0.014**	0.445 (0.240–0.824)	**0.010**
No evidence	Reference		Reference	
**Age at diagnosis**		**0.001**		**0.007**
≤60 years	Reference		Reference	
>60 years	3.248 (1.583–6.664)	**0.001**	2.854 (1.331–6.117)	**0.007**
**Sex**		0.062		0.151
Male	Reference		Reference	
Female	1.727 (0.974–3.064)	0.062	1.556 (0.851–2.846)	0.151
**Race**		0.492		0.742
White	Reference		Reference	
Black	0.271 (0.034–1.959)	0.196	0.310 (0.043–2.252)	0.247
Others^#^	0.850 (0.363–1.994)	0.709	0.989 (0.418–2.338)	0.980
**Marital status**		0.151		0.069
Married	1.575 (0.847–2.926)	0.151	1.889 (0.953–3.746)	0.069
Unmarried	Reference		Reference	
**Year of diagnosis**		**0.019**		**0.070**
2004–2009	Reference		Reference	
2010–2015	0.487 (0.267–0.889)	**0.019**	0.560 (0.299–1.048)	**0.070**
**Tumor location**		0.362		0.668
Brain	Reference		Reference	
Spine	1.642 (0.504–5.350)	0.411	0.814 (0.110–6.016)	0.840
NOS*	1.373 (0.585–3.224)	0.466	1.305 (0.471–3.611)	0.609
**Histological type**		0.498		0.706
Mature B-cell NHL	Reference		Reference	
Others^$^	1.279 (0.628–2.604)	0.498	1.180 (0.500–2.784)	0.706
**Surgery**		0.681		0.873
Yes	0.887 (0.502–1.568)	0.681	0.952 (0.520–1.744)	0.873
No evidence	Reference		Reference	

^#^Others include American Indian/Alaska Native and Asian/Pacific Islander.

*Not specific primary site of central nerve system.

^$^Others include peripheral T-cell lymphoma, anaplastic large cell lymphoma, extranodal NK-/T-cell lymphoma, blastic plasmacytoid dendritic cell neoplasm, precursor B-lymphoblastic lymphoma, precursor T-cell lymphoblastic lymphoma and not specific non-Hodgkin’s lymphoma. The bold values mean P value < 0.05.

NOS, not otherwise specific; PSM, propensity score matching; 95% CI, 95% confidence interval.

### Cause-Specific Mortality

As shown in **Table S1**, a total of 1,242 (61.5%) PCNSL patients died at the last follow-up. 1,062 (85.5%) patients died of the cancer causes, while the other 180 (14.5%) patients died of non-cancer causes. CVDs (32.8%) are the leading causes of non-cancer deaths in PCNSL patients.

### Competing Risks Regression Analysis of Cardiovascular Death in PCNSL Patients

**Figure 1:**
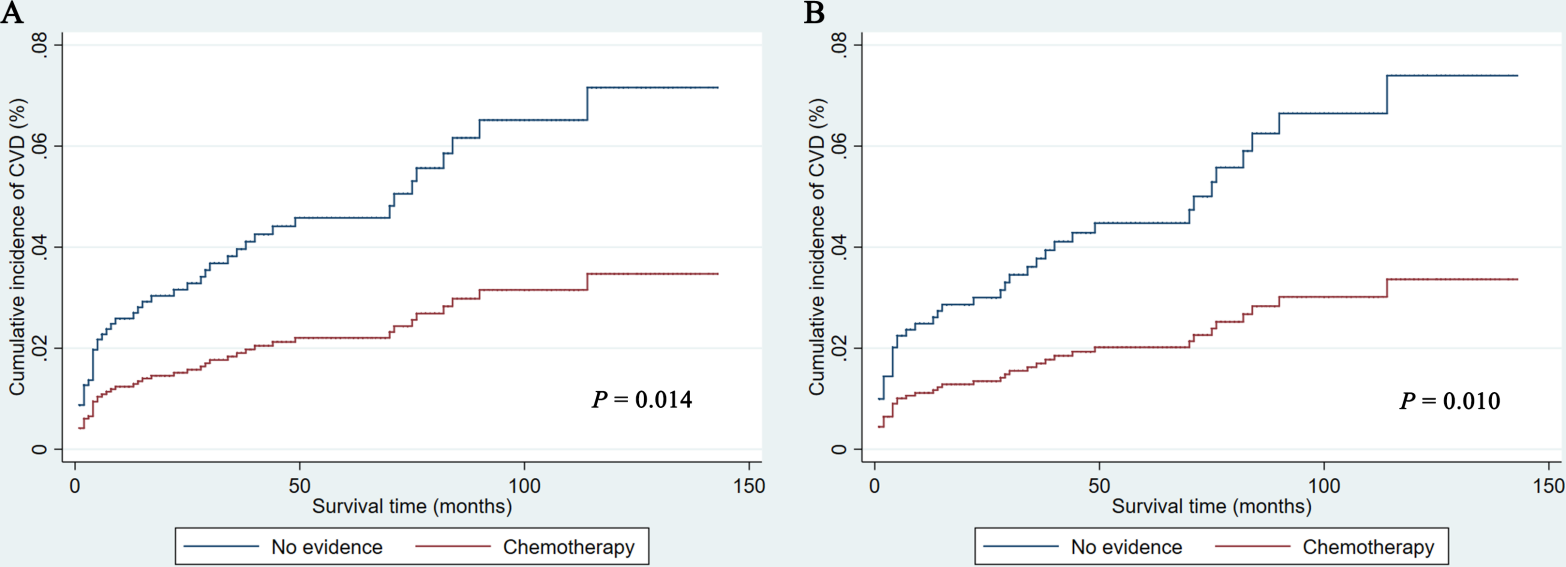
Cumulative incidence of cardiovascular death before **(A)** and after **(B)** propensity score matching in PCNSL patients. CVD, cardiovascular death.

As shown in **Figure 1**, PCNSL patients with chemotherapy were at lower CVD risk compared with no chemotherapy (before PSM: *P* = 0.014; after PSM: *P* = 0.010). As shown in **Table 2**, chemotherapy (*P* = 0.010) and age at diagnosis (*P* = 0.007) were both correlated with CVD at univariate analysis before and after PSM, while year of diagnosis (*P* = 0.019) were correlated with CVD at univariate analysis before PSM. To avoid the possibility of false-positive results, chemotherapy was further confirmed as an independent predictor of CVD at multivariate analysis before and after PSM. With the adjustment of the confounding covariates (model 1: age at diagnosis and year of diagnosis), we found a robust adjusted HR of chemotherapy after PSM [adjusted HR in model 1, 0.469 (95% CI, 0.255–0.862; *P* = 0.015)] (**Tables 3** and **S2**). Further, after adjusting for the other covariates (model 2 and model 3), the adjusted HR of chemotherapy changed indistinctively (<4%), and the CVD risk in PCNSL patients with chemotherapy decreased about 53% compared with PCNSL patients without chemotherapy (adjusted HR in model 2, 0.473 [95% CI, 0.257–0.870; *P* = 0.016]; adjusted HR in model 3, 0.446 [95% CI, 0.241–0.824; *P* = 0.010]) (**Tables 3**, **S3** and **S4**). Age at diagnosis (before PSM: *P* = 0.001; after PSM: *P* = 0.006) and year of diagnosis (before PSM: *P* = 0.009; after PSM*:P* = 0.037) were associated with CVD after adjustment at multivariate analysis (**Table S2**).

**Table 3:** Multivariate competing-risks regression analysis of cardiovascular death in PCNSLs.

Variable	Before PSM		After PSM	
	HR (95% CI)	*P* Value	HR (95% CI)	*P* Value
**Unadjusted HR**				
Chemotherapy	0.476 (0.262–0.862)	**0.014**	0.445 (0.240–0.824)	**0.010**
No evidence	Reference		Reference	
**Model 1** ^a^				
Chemotherapy	0.523 (0.290–0.943)	**0.031**	0.469 (0.255–0.862)	**0.015**
No evidence	Reference		Reference	
**Model 2** ^b^				
Chemotherapy	0.519 (0.288–0.933)	**0.028**	0.473 (0.257–0.870)	**0.016**
No evidence	Reference		Reference	
**Model 3** ^c^				
Chemotherapy	0.496 (0.274–0.898)	**0.021**	0.446 (0.241–0.824)	**0.010**
No evidence	Reference		Reference	

a In model 1, hazard ratios were adjusted for statistically significant factors according to univariate analysis (age at diagnosis and year of diagnosis).

b In model 2, hazard ratios were adjusted for all factors in model 1, plus the potential confounders included in propensity score matching (sex, tumor location, histological type and surgery).

c In model 3, hazard ratios were adjusted for all factors in model 2, plus the other demographic characteristics (race and marital status).

HR, hazard ratios; PSM, propensity score matching; 95% CI, 95% confidence interval. The bold values mean P value < 0.05.

### Competing-Risks Regression Analysis Based on Different Ages at Diagnosis

We further evaluated the impact of chemotherapy on CVD based on different ages at diagnosis (≤60 years *vs* >60 years). As shown in **Table 4**, chemotherapy was associated with lower CVD risk in PCNSL patients with age at diagnosis >60 years [before PSM: HR, 0.451 (95% CI, 0.238–0.853; *P* = 0.014); after PSM: HR, 0.390 (95% CI, 0.200–0.760; *P* = 0.006)]. Nevertheless, chemotherapy was not significantly related to CVD risk in PCNSL patients with age at diagnosis ≤60 years (before PSM: *P* = 0.746; after PSM: *P* = 0.700).

**Table 4:** Univariate competing-risks regression analysis based on different ages at diagnosis.

Age at diagnosis	Before PSM		After PSM	
	HR (95% CI)	*P* value	HR (95% CI)	*P* value
**≤60 years**				
Chemotherapy	1.407 (0.178–11.106)	0.746	1.505 (0.187–12.090)	0.700
No evidence	Reference		Reference	
**>60 years**				
Chemotherapy	0.451 (0.238–0.853)	**0.014**	0.390 (0.200–0.760)	**0.006**
No evidence	Reference		Reference	

HR, hazard ratios; PSM, propensity score matching; 95% CI, 95% confidence interval. The bold values mean P value < 0.05.

### Competing-Risks Regression Analysis Based on Different Years at Diagnosis

Given that chemotherapy regimens advanced in the last decades, we further assessed the impact of chemotherapy on CVD based on different years at diagnosis (2004–2009 *vs* 2010–2015). As shown in **Table 5**, chemotherapy was associated with lower CVD risk in PCNSL patients diagnosed at 2010 to 2015 [before PSM: HR, 0.326 (95% CI, 0.117–0.912; *P* = 0.033); after PSM: HR, 0.339 (95% CI, 0.118–0.970; *P* = 0.044)]. But chemotherapy was not significantly correlated with CVD risk in PCNSL patients diagnosed at 2004 to 2009 (before PSM: *P* = 0.153; after PSM: *P* = 0.092).

**Table 5:** Univariate competing-risks regression analysis based on different years at diagnosis.

Year of diagnosis	Before PSM		After PSM	
	HR (95% CI)	*P* Value	HR (95% CI)	*P* Value
**2004**–**2009**				
Chemotherapy	0.584 (0.280–1.220)	0.153	0.518 (0.241–1.112)	0.092
No evidence	Reference		Reference	
**2010**–**2015**				
Chemotherapy	0.326 (0.117–0.912)	**0.033**	0.339 (0.118–0.970)	**0.044**
No evidence	Reference		Reference	

HR, hazard ratios; PSM, propensity score matching; 95% CI, 95% confidence interval. The bold values mean P value < 0.05.

### The Effect of Chemotherapy on CVD Risk Between PCNSL and NCNSL

To further avoid the treatment selection bias, we evaluate the effect of chemotherapy on CVD risk between PCNSL patients with chemotherapy and NCNSL patients with chemotherapy. Baseline characteristics were showed in **Supplementary Results** and **Table S5**. As shown in **Table 6**, PCNSL patients with chemotherapy showed lower CVD risk compared with NCNSL patients with chemotherapy (before PSM: *P* = 0.006; after PSM *P* = 0.019). To avoid the possibility of false-positive results, we adjusted the confounding covariates (model 1) and other covariates (model 2), and found a robust result that the CVD risk in PCNSL patients with chemotherapy decreased about 36% compared with NCNSL patients with chemotherapy after PSM [adjusted HR in model 1, 0.636 (95% CI, 0.439–0.923; *P* = 0.017); adjusted HR in model 2, 0.640 (95% CI, 0.441–0.928; *P* = 0.019)] (**Tables 7**, **S6** and **S7**).

**Table 6:** Univariate competing-risks regression analysis of cardiovascular death between PCNSLs with chemotherapy and NCNSLs with chemotherapy.

Variable	Before PSM		After PSM	
	HR (95% CI)	*P* Value	HR (95% CI)	*P* Value
**Tumor location**				
PCNSL	0.620 (0.440–0.874)	**0.006**	0.642 (0.443–0.931)	**0.019**
NCNSL	Reference		Reference	
**Age at diagnosis**				
≤ 60 years	Reference		Reference	
> 60 years	5.568 (5.091–6.089)	**<0.001**	3.979 (2.757–5.743)	**<0.001**
**Sex**				
Male	Reference		Reference	
Female	0.962 (0.901–1.027)	0.241	1.014 (0.772–1.331)	0.923
**Race**				
White	Reference		Reference	
Black	1.106 (1.000–1.224)	0.050	1.006 (0.586–1.727)	0.983
Others^#^	0.809 (0.705–0.929)	0.003	0.828 (0.532–1.290)	0.404
**Marital status**				
Married	0.982 (0.920–1.048)	0.577	0.846 (0.642–1.116)	0.237
Unmarried	Reference		Reference	
**Year of diagnosis**				
2004–2009	Reference		Reference	
2010–2015	0.702 (0.654–0.754)	**<0.001**	0.600 (0.449–0.803)	**0.001**
**Histological type**				
Mature B-cell NHL	Reference		Reference	
Others^$^	0.419 (0.377–0.466)	**<0.001**	0.788 (0.491–1.264)	0.323
**Surgery**				
Yes	0.967 (0.891–1.050)	0.427	0.925 (0.702–1.219)	0.579
No evidence	Reference		Reference	

^#^Others include American Indian/Alaska Native and Asian/Pacific Islander.

^$^Others include peripheral T-cell lymphoma, anaplastic large cell lymphoma, extranodal NK-/T-cell lymphoma, blastic plasmacytoid dendritic cell neoplasm, precursor B-lymphoblastic lymphoma, precursor T–cell lymphoblastic lymphoma and not specific non-Hodgkin’s lymphoma.

NCNSL, non-central nervous system lymphoma; NOS, not otherwise specific; PCNSL, primary central nervous system lymphoma; PSM, propensity score matching; 95% CI, 95% confidence interval. The bold values mean P value < 0.05.

**Table 7:** The effect of chemotherapy on CVD risk between PCNSLs and NCNSLs.

Variable	Before PSM		After PSM	
	HR (95% CI)	*P* Value	HR (95% CI)	*P* Value
**Unadjusted HR**				
PCNSL	0.620 (0.440–0.874)	**0.006**	0.642 (0.443–0.931)	**0.019**
NCNSL	Reference		Reference	
**Model 1** ^a^				
PCNSL	0.579 (0.410–0.818)	**0.002**	0.636 (0.439–0.923)	**0.017**
NCNSL	Reference		Reference	
**Model 2** ^b^				
PCNSL	0.608 (0.430–0.859)	**0.005**	0.640 (0.441–0.928)	**0.019**
NCNSL	Reference		Reference	

a In model 1, hazard ratios were adjusted for statistically significant factors according to univariate analysis (age at diagnosis and year of diagnosis).

b In model 2, hazard ratios were adjusted for all factors in model 1, plus the potential confounders (sex, race, marital status, histological type, and surgery).

HR, hazard ratios; NCNSL, non-central nervous system lymphoma; PCNSL, primary central nervous system lymphoma; PSM, propensity score matching; 95% CI, 95% confidence interval. The bold values mean P value < 0.05.

## Discussion

PCNSL patients have a poor prognosis, and CVDs (32.8%) are the leading causes of non-cancer deaths in PCNSL patients. However, the CVD risk in PCNSL patients with chemotherapy is still unclear. In this multi-center retrospective study, we found that the CVD risk in PCNSL patients with chemotherapy significantly decreased by 53% and 36%, compared with those without chemotherapy and NCNSL patients with chemotherapy, respectively, which may offer new insights into the CVD risk in PCNSL patients with chemotherapy.

Chemotherapy and age at diagnosis were both significantly associated with CVD risk at univariate analysis, and were identified as independent CVD predictors for patients with PCNSL at multivariate analysis. Being different from these two variables, year of diagnosis was not statistically correlated with CVD risk at univariate analysis after PSM, but it was significantly related to CVD risk after adjustment at multivariate analysis before and after PSM. As for age at diagnosis, the CVD risk in PCNSL patients with age at diagnosis over 60 years was obviously increased by about twice compared with their counterparts, which was partly consistent with the findings of Husam et al. (20) who showed that higher cardiovascular mortality was observed in old breast cancer patients. Soisson et al. (21) also reported that endometrial cancer survivors with age less than 60 years had lower risk of cardiovascular diseases. These results emphasize the importance of CVD prevention and the monitoring for PCNSL patients over 60 years as they have a higher CVD risk.

To our best knowledge, it is the first study to show that PCNSL patients with chemotherapy were associated with lower CVD risk. Before PSM, the CVD risk in PCNSL patients with chemotherapy significantly decreased by 48%, which seems not to conform with the common perception that chemotherapy induced cardiotoxicity and increased CVD risk (6). We conducted PSM to reduce the imbalance and used multivariate analysis to adjust the confounding effects. What is more, to further reduce the treatment selection bias, especially cardiovascular comorbidities, we assessed the effect of chemotherapy on CVD risk between PCNSL and NCNSL patients with chemotherapy, which is a common and widely accepted method (18, 19). After adjustment, we obtained a consistent result that the PCNSL patients with chemotherapy were at a lower risk of CVD compared with those without chemotherapy and NCNSL patients with chemotherapy. The underlying mechanism for chemotherapy to improve cardiovascular prognosis has not been explicit yet. Hence, these results must be carefully interpreted.

These results might be related to two potential reasons: (1) bias on the baseline cardiovascular conditions, (2) effect of chemotherapeutic drugs. Firstly, in spite of all the effort we have made to avoid the potential bias, these results are still possibly attributable to the imbalance of cardiovascular conditions in the baseline characteristics, because yet we do not have any information related to the initial cardiovascular conditions of the study populations. The baseline cardiac risks of individuals without chemotherapy were probably already elevated at baseline, thus contraindicating chemotherapy. While those subjects at better cardiovascular conditions were more likely to undergo chemotherapy (22). Therefore, increased risks of CVD would be observed in the patients without chemotherapy while those received chemotherapy had lower risk of CVD.

Secondly, apart from the potential bias described above, we hypothesize that it probably attributes to the effect of chemotherapy drugs and the advance of chemotherapeutic regimens for PCNSL. Compared with those diagnosed in 2004 to 2009, the CVD risk in PCNSL patients diagnosed in 2010 to 2015 was lower, which suggested the chemotherapy for PCNSL patients has advanced in the last decades. Indeed, 2009 was an important time node related to the extensive usage of MTX (1, 5, 23). In the 1980s to 1990s, cyclophosphamide, doxorubicin, vincristine, and prednisolone (CHOP) regimen was firstly used to treat PCNSL patients, but was ineffective and induced cardiotoxicity (1, 23). In 1990s, high-dose methotrexate (HD-MTX) was introduced into the chemotherapeutic regimens and gradually proved to be effective (1, 23). By 2009, HD-MTX based chemotherapy was firstly recommended as first-line treatment for PCNSL patients (5, 23, 24). Therefore, these findings suggest that the improvement of chemotherapy on cardiovascular prognosis may be related to the extensive usage of MTX since 2009.

Interestingly, in the subgroup analysis, we further found that the improvement of chemotherapy on cardiovascular prognosis was relevant to year of diagnosis. The decreased CVD risk in PCNSL patients with chemotherapy was significant only among those diagnosed in 2010 to 2015 rather than those diagnosed in 2004 to 2009. The result may be explained by the chemotherapeutic regimens for PCNSL patients since 2009. Current chemotherapeutic regimens for PCNSL mainly involved MTX, cytarabine, carmustine, rituximab and so on (1). Some of these had been reported to be related to potential cardiotoxicity, for example, cytarabine can induce pericarditis (7, 8) and carmustine impairs cardiac function (9) while some took an uncertain effect on cardiovascular system, such as rituximab (25). Remarkably, HD-MTX, recommended as an indispensable drug in the first-line chemotherapeutic regimens, had been reported to decrease the risk for cardiovascular events (e.g. stroke, myocardial infarction, etc) and reduce cardiovascular mortality (10, 26). These results further supported that PCNSL patients with chemotherapy were associated with lower CVD risk.

What is more, as for the common perception that chemotherapy increased CVD risk in NCNSL patients, it should be noted that anthracyclines (e.g. doxorubicin, epirubicin, etc) were the backbone of chemotherapy in NCNSL (27, 28), which had been confirmed to induce cardiotoxicity and increase CVD risk (29), such as heart failure and so on (30). Our findings also supported this result that the CVD risk in PCNSL patients with chemotherapy was lower than NCNSL patients with chemotherapy. Compared with the chemotherapy regimen for NCNSL, HD-MTX was mainly used for PCNSL patients to penetrate the blood-brain barrier, and seldom used for NCNSL patients (1). Remarkably, there was less possibility for treatment selection bias (especially cardiovascular comorbidities) between PCNSL and NCNSL patients with chemotherapy, which suggested the results were reliable. It is a common and widely acceptable method that other-site tumor is used as a comparison group in the previous studies (18, 19), although the comparison between PCNSL and NCNSL may have some complex variables. All our analyses including subgroup analysis found a consistent result that PCNSL patients with chemotherapy had lower CVD risk. These results further supported our results that the lower CVD risk in PCNSL patients with chemotherapy.

In addition, although PCNSL patients over 60 years were at a high CVD risk, PCNSL patients with chemotherapy had lower CVD risk compared with those without chemotherapy. It is probably for the reason that the PCNSL patients over 60 years can benefit more from chemotherapy for their higher CVD risk. Our result indicates that the PCNSL patients over 60 years are allowed to receive chemotherapy if their basic physical conditions permit.

The underlying mechanism how MTX reduces CVD risk is as follows. MTX is a folic acid antagonist, which binds to dihydrofolate reductase preventing the conversion of tetrahydrofolate precursor to the co-factor (31). MTX takes cardioprotective effects by anti-inflammation, because inflammation plays an important role in the development of cardiovascular disease (32). MTX not only inhibits the production of proinflammatory cytokines, but also promotes the gene expression of anti-inflammation (33, 34). On the other hand, MTX also plays antiatherogenic effects by enhancing the antiatherogenic function of high-density lipoprotein (34), limiting foam cell transformation and promoting reverse cholesterol transport (35). In addition, microvascular endothelial dysfunction occurs early in the development of cardiovascular disease [e.g., stroke, coronary artery disease (CAD) and heart failure (HF)] and is worsened by inflammation. Previous research showed anti-inflammatory treatment improved microvascular function in patients with rheumatoid arthritis. Lymphomas were associated with microvascular lesions (36). Diabetic microvascular complications are characterized by functional and structural organ damage as a result of inflammation injury in the vascular system, which affects the capillaries and arterioles in the heart, retina, kidney, and nerves. In animal model of diabetes, cardiovascular inflammation was reduced with MTX (37). This indicated that microvascular dysfunction may be the potential target for MTX to reduce cardiovascular death. The clinical evidences also support the cardioprotective properties of MTX. A prospective study conducted by Choi et al. (38) reported that MTX could reduce cardiovascular mortality. A systematic review and meta-analysis further showed that MTX was related to 21% lower risk for total cardiovascular events and 18% lower risk of myocardial infarction (10). The Cardiovascular Inflammation Reduction Trial reported that low-dose MTX did not reduce atherosclerotic events in patients with stable atherosclerosis (39). However, patients with CAD seems to benefit from other anti-inflammatory drugs (e.g., colchicine). Low-doses of colchicine (LoDoCo study) decrease ischemic cardiovascular events by 23% among patients with recent myocardial infarction (40). The LoDoCo2 study (41) further confirmed this effect. These results suggest that it is necessary to screen appropriate populations (high inflammation risk) for receiving anti-inflammatory treatment besides the differences in anti-inflammatory drugs use. Indeed, recent study by Karim Labreche et al. (42) found that germline variants influenced PCNSL outcomes, and inflammation-related markers (e.g., interleukin-10) are potential targets. The underlying mechanism is still unclear and needs to be investigated further.

## Study Limitations

Several limitations should be mentioned. Firstly, retrospective studies have limitations in nature, which cannot be adequately compensated despite the adjustment for confounding covariates. Secondly, cardiovascular comorbidities are not included in the SEER database, and we were unable to further explore the impact of cardiovascular comorbidities on CVD in PCNSL patients. Recent study by Michael Brendan Cloney et al. (43) found that cardiac risk predicted systemic complications among patients with PCNSL, and the PCNSL patients with resection had higher cardiac risk, but it is still unclear whether this risk is related to surgical resection or disease itself. A case report of Cleo R van Rooijen et al. (44) found that primary cardiac lymphoma had a high risk of central nervous system recurrence, suggesting that cardiovascular system (e.g., heart, etc) may be potentially affected in PCNSL patients, which may cause various cardiovascular abnormalities and poor prognosis. Indeed, primary cardiac lymphoma patients with arrhythmia had worse prognosis compared those without (45). Also, primary cardiac lymphoma was associated with angina (46, 47). Importantly, which cardiac complications or cardiovascular diseases are involved, such as stroke (48), CAD (49), HF (50)or/and atrial fibrillation (51), etc. These cardiovascular diseases require further attention, especially HF. Thirdly, the details of the dosage, type, and duration of chemotherapeutic regimens were not recorded in SEER database. Nevertheless, it should be noted that HD-MTX based regimen is indispensable in the first-line treatment for PCNSLs since 2009 (5), which offers an insight that the improvement of chemotherapy on cardiovascular prognosis may be related to MTX. The results should be interpreted as showing an association instead of causality. Lastly, given the nature of observational study, our results should be interpreted as showing an association instead of causality. Despite these limitations, the remarkable strengths of our study are the long follow-up time, multi-center case (18 registries) and the large sample size. The underlying mechanism on MTX related to chemotherapy for cardiovascular prognosis needs to be investigated further.

## Conclusion

PCNSL patients with chemotherapy are associated with lower CVD risk compared with both those without chemotherapy and NCNSL participants with chemotherapy. Our findings provide new foundations for that chemotherapy may be the optimal treatment strategy for PCNSL patients, according to a cardiovascular risk perspective. These findings may assist clinical decision-making, and need to be further validated in prospective randomized trials.

## Data Availability Statement

Publicly available data sets were analyzed in this study. These data can be found here: https://seer.cancer.gov/.

## Author Contributions

TG: conception, study design, data collection, analysis, interpretation of results, figure design, article draft writing, and article—review and editing. ZQ and MS: study design, analysis, interpretation of results, article draft writing, and article—review and editing. JY: interpretation of results and figure design. YT and YJ: article draft writing. DY, YXL, and YFL: study design and data collection. CL: funding acquisition, interpretation of results, project administration and supervision, and article—review and editing. All authors contributed to the article and approved the submitted version.

## Funding

This study was funded by the National Natural Science Foundation of China (81100235), the Guangzhou Science and Technology Project of China (201804010214), and the Special Funds for the Cultivation of Guangdong College Students’ Scientific and Technological Innovation (“Climbing Program” Special Funds) (pdjh2020b0485).

## Conflict of Interest

The authors declare that the research was conducted in the absence of any commercial or financial relationships that could be construed as a potential conflict of interest.

## Abbreviations

CAD, Coronary artery disease; CHOP, Cyclophosphamide doxorubicin vincristine and prednisolone; CI, Confidence interval; CT, Chemotherapy; CVD, Cardiovascular death; HD-MTX, High dose- methotrexate; HR, Hazard ratio; NCNSL, Non-central nervous system lymphoma; NHL, Non-Hodgkin lymphoma; NOS, Not otherwise specific; PCNSL, Primary central nervous system lymphoma; PSM, Propensity score matching; MTX, Methotrexate; SEER, The Surveillance Epidemiology and End Result database.
