# Supplementary material for: Cardiovascular Death Risk in Primary Central Nervous System Lymphoma Patients Treated With Chemotherapy: A Registry-Based Cohort Study
Source: Front Oncol. 2021 May 11;11:641955. doi: 10.3389/fonc.2021.641955 (PMC8147725; doi:10.3389/fonc.2021.641955)
Supplement: Supplementary file 1 [file Table_1.docx]

**Supplementary Materials**

**Supplementary methods**

**Study population**

Non-central nervous system lymphoma (NCNSL) was defined as patients with a first primary malignant non-Hodgkin’s lymphoma (NHL) outside central nerve system. The selection criteria were as follow: (1) case selection {Site and Morphology. Lymphoma subtype recode/WHO 2008} = ‘2 Non-Hodgkin Lymphoma’; (2) case selection {Site and Morphology. Diagnostic Confirmation} = ‘Positive histology’; (3) participants who received chemotherapy; (4) year of diagnosis = ‘2004-2015’. The exclusion criteria were as follows: (1) participants who received radiotherapy; (2) participants with multiple primary tumors; (3) either autopsy only or death certificate only; (4) primary site at central nervous system; (5) unknown race; (6) unknown marital status. Finally, a total of 88,613 NCNSL participants with chemotherapy were extracted according to the selection and exclusion criteria.

**Statistical analysis**

A 1:3 propensity score matching (PSM) was to reduce the imbalance between primary central nervous system lymphoma (PCNSL) and NCNSL patients with chemotherapy. The variables were enrolled into the propensity score calculating: age at diagnosis, sex, race, marital status, year of diagnosis, histological type and surgery. Matching was conducted using nearest-neighbor algorithm with caliper width of 0.01. A P value of larger than 0.05 for the above covariates was regarded as acceptable balance.

**Supplementary results**

**Baseline characteristics between PCNSL patients with chemotherapy and NCNSL patients with chemotherapy**

As shown in Table S5, there were 1,493 PCNSL patients with chemotherapy and 88,613 NCNSL patients with chemotherapy. Before PSM, PCNSL group was prone to had less younger, male, white, married patients and had more Mature B-cell NHL patients, patients with surgery and patients diagnosed at 2010-2015 compared to NCNSL group. After the 1:3 PSM, 1,493 PCNSL patients with chemotherapy and 4,479 NCNSL patients with chemotherapy were allocated to the matched cohort, and the confounding covariates (age at diagnosis, sex, year of diagnosis, tumor location, histological type and surgery) were well balanced between the chemotherapy and no chemotherapy groups (Table S5).

**Supplementary Table captions**

**Table S1.** The cause-specific mortality in in PCNSLs

*Cardiovascular disease includes cerebrovascular diseases, diseases of heart and hypertension without heart disease and;

^†^Other Infectious and Parasitic Diseases including HIV;

^#^Respiratory disease includes pneumonia, influenza and chronic obstructive pulmonary disease and allied cond;

**^∆^** Digestive disease includes chronic liver disease, cirrhosis, stomach and duodenal ulcers.

**Table S2.** Multivariate competing-risks regression analysis of cardiovascular death (Model 1)

Abbreviation: HR, hazard ratios; NOS, not otherwise specific; PSM, propensity score matching.

**Table S3.** Multivariate competing-risks regression analysis of cardiovascular death (Model 2)

*Not specific primary site of central nerve system.

^$^Others includs peripheral T-cell lymphoma, anaplastic large cell lymphoma, extranodal NK-/T-cell lymphoma, blastic plasmacytoid dendritic cell neoplasm, precursor B-lymphoblastic lymphoma, precursor T--cell lymphoblastic lymphoma and not specific non-Hodgkin’s lymphoma.

Abbreviation: HR, hazard ratios; NOS, not otherwise specific; PSM, propensity score matching.

**Table S4.** Multivariate competing-risks regression analysis of cardiovascular death (Model 3)

^#^Others includes American Indian/ Alaska Native and Asian/Pacific Islander.

*Not specific primary site of central nerve system.

^$^Others includs peripheral T-cell lymphoma, anaplastic large cell lymphoma, extranodal NK-/T-cell lymphoma, blastic plasmacytoid dendritic cell neoplasm, precursor B-lymphoblastic lymphoma, precursor T--cell lymphoblastic lymphoma and not specific non-Hodgkin’s lymphoma.

Abbreviation: HR, hazard ratios; NOS, not otherwise specific; PSM, propensity score matching.

**Table S5.** Baseline characteristics between PCNSLs with chemotherapy and NCNSLs with chemotherapy

^#^Others includes American Indian/ Alaska Native and Asian/Pacific Islander.

^$^Others includs peripheral T-cell lymphoma, anaplastic large cell lymphoma, extranodal NK-/T-cell lymphoma, blastic plasmacytoid dendritic cell neoplasm, precursor B-lymphoblastic lymphoma, precursor T--cell lymphoblastic lymphoma and not specific non-Hodgkin’s lymphoma.

Abbreviation: CT, Chemotherapy; CVD, cardiovascular death; NCNSL, Non-central nervous system lymphoma; NOS, not otherwise specific; PCNSL, primary central nervous system lymphoma; PSM, propensity score matching.

**Table S6.** Multivariate competing-risks regression analysis of cardiovascular death between PCNSLs with chemotherapy and NCNSLs with chemotherapy (Model 1)

Abbreviation: HR, hazard ratios; NCNSL, Non-central nervous system lymphoma; NOS, not otherwise specific; PCNSL, primary central nervous system lymphoma; PSM, propensity score matching.

**Table S7.** Multivariate competing-risks regression analysis of cardiovascular death between PCNSLs with chemotherapy and NCNSLs with chemotherapy (Model 2)

^#^Others includes American Indian/ Alaska Native and Asian/Pacific Islander.

^$^Others includs peripheral T-cell lymphoma, anaplastic large cell lymphoma, extranodal NK-/T-cell lymphoma, blastic plasmacytoid dendritic cell neoplasm, precursor B-lymphoblastic lymphoma, precursor T--cell lymphoblastic lymphoma and not specific non-Hodgkin’s lymphoma.

Abbreviation: HR, hazard ratios; NCNSL, Non-central nervous system lymphoma; NOS, not otherwise specific; PCNSL, primary central nervous system lymphoma; PSM, propensity score matching.

**Table S1. The cause-specific mortality in in PCNSLs**

| **Cause of death** | **N** | **Percentage in overall death** | **Percentage in non-cancer related death** |
| --- | --- | --- | --- |
| Overall death | 1242 | 100.0% | — |
| Total cancer causes of death | 1062 | 85.5% | — |
| Non-cancer related death | 180 | 14.5% | 100.0% |
| Cardiovascular disease* | 59 | 4.8% | 32.8% |
| Respiratory disease^#^ | 13 | 1.0% | 7.2% |
| Infectious and Parasitic Diseases ^†^ | 10 | 0.8% | 5.6% |
| Accidents and adverse effects | 7 | 0.6% | 3.9% |
| Alzheimer | 7 | 0.6% | 3.9% |
| Nephritis, nephrotic syndrome and nephrosis | 4 | 0.3% | 2.2% |
| Septicemia | 4 | 0.3% | 2.2% |
| Digestive disease**^∆^** | 3 | 0.2% | 1.7% |
| Symptoms, signs and ill-defined conditions | 3 | 0.2% | 1.7% |
| Diabetes mellitus | 2 | 0.2% | 1.1% |
| Suicide and self-inflicted injury | 1 | 0.1% | 0.6% |
| Other cause of death | 45 | 3.6% | 25.0% |
| Unknown reason | 22 | 1.8% | 12.2% |

*Cardiovascular disease includes cerebrovascular diseases, diseases of heart and hypertension without heart disease and;

^†^Other Infectious and Parasitic Diseases including HIV;

^#^Respiratory disease includes pneumonia, influenza and chronic obstructive pulmonary disease and allied cond;

**^∆^** Digestive disease includes chronic liver disease, cirrhosis, stomach and duodenal ulcers.

**Table S2.** **Multivariate competing-risks regression analysis of cardiovascular death in PCNSLs (Model 1)**

| **Variable** | **Before PSM** | |  | **After PSM** | |
| --- | --- | --- | --- | --- | --- |
|  | **HR (95% CI)** | ***P* Value** |  | **HR (95% CI)** | ***P* Value** |
| **Chemotherapy** |  |  |  |  |  |
| Yes | 0.523 (0.290-0.943) | 0.031 |  | 0.469 (0.255-0.862) | 0.015 |
| No evidence | Reference |  |  | Reference |  |
| **Age at diagnosis** |  |  |  |  |  |
| ≤60 years | Reference |  |  | Reference |  |
| >60 years | 3.267 (1.610-6.630) | 0.001 |  | 2.878 (1.358-6.097) | 0.006 |
| **Year of diagnosis** |  |  |  |  |  |
| 2004-2009 | Reference |  |  | Reference |  |
| 2010-2015 | 0.453 (0.250-0.823) | 0.009 |  | 0.516 (0.277-0.962) | 0.037 |

Abbreviation: HR, hazard ratios; NOS, not otherwise specific; PSM, propensity score matching.

**Table S3. Multivariate competing-risks regression analysis of cardiovascular death in PCNSLs (Model 2)**

| **Variable** | **Before PSM** | |  | **After PSM** | |
| --- | --- | --- | --- | --- | --- |
|  | **HR (95% CI)** | ***P* Value** |  | **HR (95% CI)** | ***P* Value** |
| **Chemotherapy** |  |  |  |  |  |
| Yes | 0.519 (0.288-0.933) | 0.028 |  | 0.473 (0.257-0.870) | 0.016 |
| No evidence | Reference |  |  | Reference |  |
| **Age at diagnosis** |  |  |  |  |  |
| ≤60 years | Reference |  |  | Reference |  |
| >60 years | 3.328 (1.590-6.966) | 0.001 |  | 2.826 (1.313-6.086) | 0.008 |
| **Sex** |  |  |  |  |  |
| Male | Reference |  |  | Reference |  |
| Female | 1.597 (0.906-2.817) | 0.106 |  | 1.380 (0.772-2.466) | 0.277 |
| **Year of diagnosis** |  |  |  |  |  |
| 2004-2009 | Reference |  |  | Reference |  |
| 2010-2015 | 0.464 (0.256-0.842) | 0.012 |  | 0.519 (0.279-0.967) | 0.039 |
| **Tumor location** |  |  |  |  |  |
| Brain | Reference |  |  | Reference |  |
| Spine | 2.383 (0.724-7.841) | 0.153 |  | 1.065 (0.144-7.887) | 0.951 |
| NOS* | 1.516 (0.648-3.549) | 0.337 |  | 1.447 (0.511-4.097) | 0.486 |
| **Histological type** |  | 0.149 |  |  | 0.703 |
| Mature B-cell NHL | Reference |  |  | Reference |  |
| Others^$^ | 1.687 (0.829-3.437) | 0.149 |  | 1.183 (0.498-2.808) | 0.703 |
| **Surgery** |  | 0.702 |  |  | 0.810 |
| Yes | 0.894 (0.504-1.587) | 0.702 |  | 0.928 (0.510-1.693) | 0.810 |
| No evidence | Reference |  |  | Reference |  |

*Not specific primary site of central nerve system.

^$^Others includs peripheral T-cell lymphoma, anaplastic large cell lymphoma, extranodal NK-/T-cell lymphoma, blastic plasmacytoid dendritic cell neoplasm, precursor B-lymphoblastic lymphoma, precursor T--cell lymphoblastic lymphoma and not specific non-Hodgkin’s lymphoma.

Abbreviation: HR, hazard ratios; NOS, not otherwise specific; PSM, propensity score matching.

**Table S4. Multivariate competing-risks regression analysis of cardiovascular death in PCNSLs (Model 3)**

| **Variable** | **Before PSM** | |  | **After PSM** | |
| --- | --- | --- | --- | --- | --- |
|  | **HR (95% CI)** | ***P* Value** |  | **HR (95% CI)** | ***P* Value** |
| **Chemotherapy** |  |  |  |  |  |
| Yes | 0.496 (0.274-0.898) | 0.021 |  | 0.446 (0.241-0.824) | 0.010 |
| No evidence | Reference |  |  | Reference |  |
| **Age at diagnosis** |  |  |  |  |  |
| ≤60 years | Reference |  |  | Reference |  |
| >60 years | 2.991 (1.457-6.139) | 0.003 |  | 2.517 (1.196-5.298) | 0.015 |
| **Sex** |  |  |  |  |  |
| Male | Reference |  |  | Reference |  |
| Female | 1.760 (1.002-3.091) | 0.049 |  | 1.583 (0.882-2.841) | 0.124 |
| **Race** |  |  |  |  |  |
| White | Reference |  |  | Reference |  |
| Black | 0.417 (0.060-2.902) | 0.377 |  | 0.482 (0.069-3.379) | 0.463 |
| Others^#^ | 0.877 (0.360-2.134) | 0.773 |  | 0.968 (0.391-2.396) | 0.944 |
| **Marital status** |  |  |  |  |  |
| Married | 1.623 (0.862-3.055) | 0.133 |  | 1.925 (0.954-3.882) | 0.067 |
| Unmarried | Reference |  |  | Reference |  |
| **Year of diagnosis** |  |  |  |  |  |
| 2004-2009 | Reference |  |  | Reference |  |
| 2010-2015 | 0.478 (0.263-0.870) | 0.016 |  | 0.536 (0.287-1.002) | 0.051 |
| **Tumor location** |  |  |  |  |  |
| Brain | Reference |  |  | Reference |  |
| Spine | 2.502 (0.745-8.402) | 0.138 |  | 1.058 (0.140-7.990) | 0.956 |
| NOS* | 1.498 (0.640-3.507) | 0.351 |  | 1.437 (0.506-4.081) | 0.496 |
| **Histological type** |  | 0.174 |  |  | 0.788 |
| Mature B-cell NHL | Reference |  |  | Reference |  |
| Others^$^ | 1.644 (0.803-3.363) | 0.174 |  | 1.126 (0.475-2.667) | 0.788 |
| **Surgery** |  | 0.717 |  |  | 0.819 |
| Yes | 0.899 (0.506-1.598) | 0.717 |  | 0.932 (0.508-1.708) | 0.819 |
| No evidence | Reference |  |  | Reference |  |

^#^Others includes American Indian/ Alaska Native and Asian/Pacific Islander.

*Not specific primary site of central nerve system.

^$^Others includs peripheral T-cell lymphoma, anaplastic large cell lymphoma, extranodal NK-/T-cell lymphoma, blastic plasmacytoid dendritic cell neoplasm, precursor B-lymphoblastic lymphoma, precursor T--cell lymphoblastic lymphoma and not specific non-Hodgkin’s lymphoma.

Abbreviation: HR, hazard ratios; NOS, not otherwise specific; PSM, propensity score matching.

**Table S5. Baseline characteristics between PCNSLs with chemotherapy and NCNSLs with chemotherapy**

| **Variable** | **Before PSM (N/%)** | | | | | | | |  | **After PSM (N/%)** | | | | | |
| --- | --- | --- | --- | --- | --- | --- | --- | --- | --- | --- | --- | --- | --- | --- | --- |
|  | **NCNSL** | **PCNSL** | | ***P* value** | | | | |  | **NCNSL** | **PCNSL** | | ***P* value** | | |
| **N** | 88,613 | 1,493 | |  | | | | |  | 4,479 | 1,493 | |  | | |
| **Age at diagnosis** |  |  | | < 0.001 | | | | |  |  |  | | 0.988 | | |
| ≤60 years | 43,618 (49.2) | | 642(43.0) |  | | | | |  | 1,927 (43.0) | | 642 (43.0) | |  | |
| >60 years | 44,995 (50.8) | | 851 (57.0) | | |  | | |  | 2,552 (57.0) | | 851 (57.0) | |  | |
| **Sex** |  |  | | 0.001 | | | | |  |  | |  | | 0.411 | |
| Male | 50,282 (56.7) | | 784 (52.5) | | |  | | |  | 2,297 (51.3) | | 784 (52.5) | |  | |
| Female | 38,331 (43.3) | | 709 (47.5) | | |  | | |  | 2,182 (48.7) | | 709 (47.5) | |  | |
| **Race** |  | |  | | < 0.001 | | | |  |  | |  | | 0.613 | |
| White | 72,451 (81.8) | | 1,207 (80.8) | | | |  | |  | 3,580 (79.9) | | 1,207 (80.8) | | |  |
| Black | 9,565 (10.8) | | 90 (6.0) | | |  | | |  | 301 (6.7) | | 90 (6.0) | |  | |
| Others^#^ | 6,597 (7.4) | | 196 (13.1) | | |  | | |  | 598 (13.4) | | 196 (13.1) | |  | |
| **Marital status** |  | |  | | < 0.001 | | | |  |  | |  | | 0.689 | |
| Married | 40,506 (45.7) | | 562 (37.6) | | |  | | |  | 1,712 (38.2) | | 562 (37.6) | |  | |
| Unmarried | 48,107 (54.3) | | 931 (62.4) | | |  | | |  | 2,767 (61.8) | | 931 (62.4) | |  | |
| **Year of diagnosis** |  | |  | | < 0.001 | | | |  |  | |  | | 0.185 | |
| 2004-2009 | 46,460 (52.4) | | 675 (45.2) | |  | | | |  | 1,937 (43.2) | | 675 (45.2) | |  | |
| 2010-2015 | 42,153 (47.6) | | 818 (54.8) | |  | | | |  | 2,542 (56.8) | | 818 (54.8) | |  | |
| **Histological Type** |  | |  | | < 0.001 | | | |  |  | |  | | 0.382 | |
| Mature B-cell NHL | 69,142 (78.0) | | 1,334 (89.4) | | | |  | |  | 3,965 (88.5) | | 1,334 (89.4) | | |  |
| Other^$^ | 19,471 (22.0) | | 159 (10.6) | |  | | | |  | 514 (11.5) | | 159 (10.6) | |  | |
| **Surgery** |  | |  | | < 0.001 | | | |  |  | |  | | 0.716 | |
| Yes | 73,129 (82.5) | | 863 (57.8) | | |  | | |  | 2,613 (58.3) | | 863 (57.8) | |  | |
| No evidence | 15,484 (17.5) | | 630 (42.2) | | |  | | |  | 1,866 (41.7) | | 630 (42.2) | |  | |
| **CVD** |  | |  | < 0.001 | | | | |  |  | |  | | 0.005 | |
| No | 84,911 (95.8) | | 1,458 (97.7) | | | | |  |  | 4,304 (96.1) | | 1,458 (97.7) | | |  |
| Yes | 3,702 (4.2) | 35 (2.3) | |  | | | | |  | 175 (3.9) | | 35 (2.3) | |  | |

^#^Others includes American Indian/ Alaska Native and Asian/Pacific Islander.

^$^Others includs peripheral T-cell lymphoma, anaplastic large cell lymphoma, extranodal NK-/T-cell lymphoma, blastic plasmacytoid dendritic cell neoplasm, precursor B-lymphoblastic lymphoma, precursor T--cell lymphoblastic lymphoma and not specific non-Hodgkin’s lymphoma.

Abbreviation: CT, Chemotherapy; CVD, cardiovascular death; NCNSL, Non-central nervous system lymphoma; NOS, not otherwise specific; PCNSL, primary central nervous system lymphoma; PSM, propensity score matching.

**Table S6. Multivariate competing-risks regression analysis of cardiovascular death between PCNSLs with chemotherapy and NCNSLs with chemotherapy (Model 1)**

| **Variable** | **Before PSM** | |  | **After PSM** | |
| --- | --- | --- | --- | --- | --- |
|  | **HR (95% CI)** | ***P* Value** |  | **HR (95% CI)** | ***P* Value** |
| **Tumor location** |  |  |  |  |  |
| PCNSL | 0.579 (0.410-0.818) | 0.002 |  | 0.636 (0.439-0.923) | 0.017 |
| NCNSL | Reference |  |  | Reference |  |
| **Age at diagnosis** |  |  |  |  |  |
| ≤60 years | Reference |  |  | Reference |  |
| >60 years | 5.726 (5.234-6.263) | < 0.001 |  | 4.111 (2.848-5.933) | < 0.001 |
| **Year of diagnosis** |  |  |  |  |  |
| 2004-2009 | Reference |  |  | Reference |  |
| 2010-2015 | 0.639 (0.596-0.686) | < 0.001 |  | 0.558 (0.418-0.745) | < 0.001 |

Abbreviation: HR, hazard ratios; NCNSL, Non-central nervous system lymphoma; NOS, not otherwise specific; PCNSL, primary central nervous system lymphoma; PSM, propensity score matching.

**Table S7. Multivariate competing-risks regression analysis of cardiovascular death between PCNSLs with chemotherapy and NCNSLs with chemotherapy (Model 2)**

| **Variable** | **Before PSM** | |  | **After PSM** | |
| --- | --- | --- | --- | --- | --- |
|  | **HR (95% CI)** | ***P* Value** |  | **HR (95% CI)** | ***P* Value** |
| **Tumor location** |  |  |  |  |  |
| PCNSL | 0.608 (0.430-0.859) | 0.005 |  | 0.640 (0.441-0.928) | 0.019 |
| NCNSL | Reference |  |  | Reference |  |
| **Age at diagnosis** |  |  |  |  |  |
| ≤60 years | Reference |  |  | Reference |  |
| >60 years | 5.697 (5.181-6.264) | < 0.001 |  | 4.333 (2.983-6.294) | < 0.001 |
| **Sex** |  |  |  |  |  |
| Male | Reference |  |  | Reference |  |
| Female | 0.791 (0.740-0.846) | < 0.001 |  | 0.864 (0.661-1.130) | 0.286 |
| **Race** |  |  |  |  |  |
| White | Reference |  |  | Reference |  |
| Black | 1.268 (1.145-1.405) | < 0.001 |  | 1.352 (0.785-2.329) | 0.277 |
| Others^#^ | 0.899 (0.783-1.032) | 0.131 |  | 0.892 (0.569-1.397) | 0.617 |
| **Marital status** |  |  |  |  |  |
| Married | 0.737 (0.689-0.789) | < 0.001 |  | 0.744 (0.564-0.982) | 0.037 |
| Unmarried | Reference |  |  | Reference |  |
| **Year of diagnosis** |  |  |  |  |  |
| 2004-2009 | Reference |  |  | Reference |  |
| 2010-2015 | 0.629 (0.586-0.675) | < 0.001 |  | 0.549 (0.410-0.734) | < 0.001 |
| **Histological type** |  |  |  |  |  |
| Mature B-cell NHL | Reference |  |  | Reference |  |
| Others^$^ | 0.651 (0.582-0.728) | < 0.001 |  | 0.851 (0.523-1.386) | 0.518 |
| **Surgery** |  |  |  |  |  |
| Yes | 0.934 (0.859-1.015) | 0.108 |  | 0.926 (0.702-1.222) | 0.585 |
| No evidence | Reference |  |  | Reference |  |

^#^Others includes American Indian/ Alaska Native and Asian/Pacific Islander.

^$^Others includs peripheral T-cell lymphoma, anaplastic large cell lymphoma, extranodal NK-/T-cell lymphoma, blastic plasmacytoid dendritic cell neoplasm, precursor B-lymphoblastic lymphoma, precursor T--cell lymphoblastic lymphoma and not specific non-Hodgkin’s lymphoma.

Abbreviation: HR, hazard ratios; NCNSL, Non-central nervous system lymphoma; NOS, not otherwise specific; PCNSL, primary central nervous system lymphoma; PSM, propensity score matching.
